# Effects of episodic future thinking on reinforcement pathology during smoking cessation treatment among individuals with substance use disorders

**DOI:** 10.1007/s00213-021-06057-6

**Published:** 2022-01-12

**Authors:** Ángel García-Pérez, Gema Aonso-Diego, Sara Weidberg, Roberto Secades-Villa

**Affiliations:** Department of Psychology, University of Oviedo, Plaza Feijoo s/n, 33003 Oviedo, Spain

**Keywords:** Smoking, Delay discounting, Cigarette demand, Reinforcer pathology, Episodic future thinking, CPT

## Abstract

**Rationale:**

Reinforcer pathology (RP) is a theoretical model based on two processes: delay discounting (DD) and drug demand. Given that RP has been shown to have a predictive value on smoking behaviors, several studies have explored which interventions can reduce RP. Consistent with the RP framework, episodic future thinking (EFT) has shown effects on treatment outcomes and RP processes. The vast majority of studies that assess the effects of EFT on RP consist of experimental studies, and no previous research has tested these effects in a clinical sample of smokers.

**Objectives:**

The primary aim of this study was to assess the effects of EFT on RP throughout the course of a smoking cessation intervention in smokers with substance use disorders (SUDs).

**Methods:**

Participants were randomized to cognitive behavior therapy (CBT) + EFT (*n* = 39) or CBT + EFT + contingency management (*n* = 33). Cotinine, frequency of EFT practices, cigarette purchase task (CPT), and DD were evaluated in treatment sessions. Mixed-effects model repeated measures analysis was used to explore DD and CPT in-treatment changes as a function of EFT practices and cotinine levels.

**Results:**

Greater practice of the EFT component significantly reduced cigarette demand (*p* < .020) as well as DD (*p* = .003). Additionally, a greater reduction in cotinine levels coupled with greater EFT practice led to a greater decrease in cigarette demand (*p* < .014).

**Conclusions:**

EFT reduced the two facets of RP in treatment-seeking smokers with SUDs.

## Introduction

Reinforcer pathology (RP) is a novel theoretical model in the field of addictions that allows us to understand substance use disorders (SUDs) based on two processes: (1) delay discounting (DD) and (2) drug demand (Bickel et al. 2014, 2019, 2020). DD refers to the observation that the value of a delayed reinforcer is discounted (reduced in value) compared to the value of an immediate reinforcer. In the context of RP, DD would involve the rapid devaluation of delayed and bigger consequences (e.g., health benefits of smoking cessation or losing weight) in preference of present and smaller rewards (e.g., smoking or overeating). On the other hand, drug demand consists of the evaluation of the motivation to procure and consume drugs. This is usually evaluated through the demand curve for a drug, whereby the consumption of a substance changes as its price increases. In the context of RP, drug demand would involve the overvaluation of a given substance compared to other reinforcers in a person’s life (e.g., money).

Numerous studies have found that an elevated RP among smokers, that is, a high DD and/or a high cigarette demand, is related to greater cigarette consumption (González-Roz et al. 2019; Reynolds 2004) and nicotine dependence (Amlung and MacKillop 2014; Cassidy et al. 2020; González-Roz et al. 2019), as well as to lower abstinence rates after receiving smoking cessation treatment (Harvanko et al. 2019; Mackillop et al. 2016; Miglin et al. 2017; Murphy et al. 2017; Secades-Villa et al. 2016) and a higher risk of relapse (García-Pérez et al. 2021).

Given that RP has been shown to have a predictive value on smoking behaviors, in recent years, several studies have sought to determine which interventions can reduce DD and cigarette demand (Scholten et al. 2019). Additionally, there are several interventions for smoking cessation (i.e., contingency management (CM), cognitive-behavioral therapy (CBT), varenicline, and low-nicotine cigarettes) that—despite not being primarily aimed at decreasing the two processes of RP—reduce DD (García-Pérez et al. 2020; Secades-Villa et al. 2014; Weidberg et al. 2015; Yi et al. 2008) and/or cigarette demand (Green and Ray 2018; Higgins et al. 2018; McClure et al. 2012; Murphy et al. 2017; Schlienz et al. 2014; Smith et al. 2017; Weidberg et al. 2018).

Consistent with the RP framework, episodic future thinking (EFT) is a novel treatment that consists of imagining future events in order to forego immediate pleasures in pursuit of longer-term aims (Hollis-Hansen et al. 2019; Rung and Epstein 2020; Schacter et al. 2017). EFT has shown effects on substance use, such as reduction of tobacco use (Chiou and Wu 2017; Stein et al. 2016), and reduction of alcohol use (Voss et al. 2021). Additionally, in substance users, EFT has been shown to reduce DD rates (Athamneh et al. 2021; Bulley and Gullo 2017; Chiou and Wu 2017; Forster et al. 2021; Mellis et al. 2019; Patel and Amlung 2020; Snider et al. 2016; Sofis et al. 2020; Stein et al. 2016, 2018) and drug demand indices (Athamneh et al. 2021; Bulley and Gullo 2017; Patel and Amlung 2020; Snider et al. 2016, 2018; Voss et al. 2021).

Despite this body of knowledge, important questions remain regarding the effectiveness of EFT. For example, previous research has yielded mixed results regarding the effect of EFT on RP in this specific population, mainly due to the limitations of these individuals in neuropsychological processes involving executive control, episodic memory, and decision-making (D’Argembeau et al. 2006; El Haj et al. 2019; Hallford et al. 2018; Mercuri et al. 2015, 2016, 2018; Moustafa et al. 2018). Also, the vast majority of studies that assess the effects of EFT on RP consist of experimental studies conducted in highly controlled laboratory settings (see, e.g., Chiou and Wu 2017; Stein et al. 2016, 2018), and no previous research has tested these effects longitudinally in a clinical sample of substance users, including smokers with SUDs.

The present study is derived from a randomized controlled trial (RCT) comparing the efficacy of CBT + EFT vs CBT + EFT + CM for smoking cessation in smokers with SUD, the results of which at end-of-treatment showed that the treatment group that included CM presented better smoking cessation outcomes (Aonso-Diego et al., 2021). According to the previous literature, both CBT and CM have been shown to be useful in reducing both DD and cigarette demand (García-Pérez et al. 2020; Secades-Villa et al. 2014; Weidberg et al. 2015, 2018; Yi et al. 2008). Therefore, with regard to the current study, combining these two components together with EFT could be remarkably beneficial for modifying RP.

The primary aim of this study was to assess the effects of EFT on the two dimensions of RP (i.e., DD and cigarette demand) throughout the course of a smoking cessation intervention in a sample of individuals with SUDs. The secondary objective was to examine the impact of tobacco use reduction and the two treatment conditions (CBT + EFT vs. CBT + EFT + CM) on RP.

## Material and methods

### Participants

This secondary analysis is derived from a randomized controlled trial (Clinical Trials-Gov Identifier: NCT03551704) aimed at the treatment of smoking in SUD individuals (Aonso-Diego et al. 2021), which was approved by the research ethics committee of the Principality of Asturias (n°144/16).

Participants were 72 treatment-seeking smokers with SUDs, and the inclusion criteria were (1) being at least 18 years old, (2) smoking at least 10 cigarettes per day for the last year, and (3) being in outpatient substance use treatment. Exclusion criteria were (1) not being able to attend the full treatment, (2) having severe mental disorders (i.e., active psychotic disorder and/or suicidal ideation), and (3) receiving another smoking cessation treatment (either psychological or pharmacological) at time of intake.

### Interventions

Participants provided informed consent and were randomized to each of the following intervention conditions: CBT + EFT (*n* = 38) and CBT + EFT + CM (*n* = 34). Table 1 shows the sociodemographic and clinical characteristics of the sample.

**Table 1:** Baseline participant characteristics

	Total (*n* = 72)
Age	44.46 ± 10.25
Sex (male)^a^	52 (72.22%)
Marital status (married)^a^	18 (25%)
Working status (employed)^a^	24 (33.33%)
Educational level (< high school)^a^	33 (45.83%)
Monthly income (€)	1314.73 ± 1329.94
Tobacco use-related variables	
CPD	19.96 ± 9.72
Years of regular use	26.24 ± 11.26
Previous quit attempts	1.36 ± 1.45
CO^a^	21.71 ± 15.55
Urine cotinine^a^	2181.35 ± 1464.01
Substance use-related variables	
Primary substance^a^	
Cocaine	27 (37.50%)
Alcohol	25 (34.72%)
Opioids	11 (15.27%)
Other^b^	9 (12.50%)
Secondary substance^a^	
None	46 (63.88%)
Cocaine	5 (6.94%)
Alcohol	10 (13.88%)
Cannabis	8 (11.11%)
Opioids	2 (2.77%)
Benzodiazepines	1 (1.38%)
Days of primary substance abstinence	274.60 ± 409.62
Days on substance use treatment	351.71 ± 633.89
BDI-II	14.34 ± 11.42
UPPS-P	
Lack of perseverance	7.58 ± 2.35
Lack of premeditation	8.01 ± 2.18
Positive urgency	10.96 ± 2.48
Negative urgency	11.54 ± 2.76
Sensation seeking	9.86 ± 2.94
DD (AUC)	0.671 ± 0.249
CPT index	
Intensity	21.24 ± 12.01
Breakpoint	11.80 ± 21.95
Omax	14.21 ± 16.27
Pmax	4.89 ± 10.43
Elasticity	0.023 ± 0.094

^a^frequency (percentage); ^b^include cannabis, ketamine, GHB, and benzodiazepines. *CPD*, cigarettes per day; *CO*, carbon monoxide in parts per million; *BDI-II*, Beck depression inventory, second edition; *UPPS-P*, impulsive behavior scale; *DD*, delay discounting; *AUC*, area under curve; *CPT*, cigarette purchase task

**Fig. 1:**
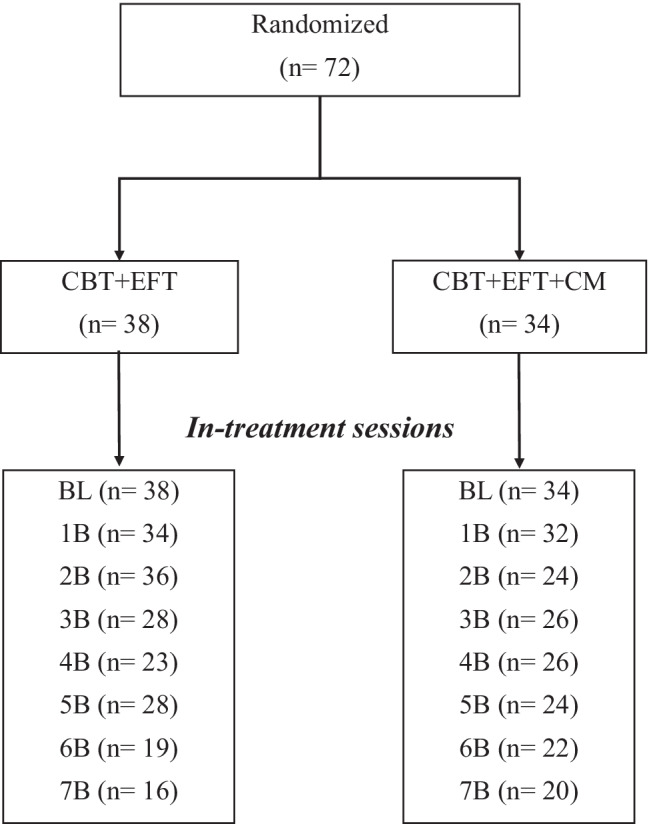
Retention of participants for each group intervention. No differences were found in participant retention between the treatment groups (*χ*^2^(7) = 3.522; *p* = .833). *BL*, baseline

All interventions were led by doctoral or master’s level psychologists with experience in the treatment of smoking. The treatments were designed in a group format, with a maximum of four participants per group. The intervention lasted eight weeks, and participants attended one weekly therapy session (session A) and one-midweek session (session B) to collect information on biochemical data and other clinical variables. Figure 1 shows the retention of the participants throughout the treatment.

The CBT protocol for smoking cessation consisted of the following components: gradual fading of nicotine intake (20% per week), self-report of tobacco use, psychoeducation, stimulus control, coping skills training to help patients to effectively manage their smoking withdrawal symptoms, problem-solving, and relapse prevention strategies. The quit date was set at 48 h before the start of the sixth session.

Following prior recommendations (Hollis-Hansen et al. 2019; Snider et al. 2016), EFT was implemented from the first session. The aim of this component was to decrease patients’ impulsive choice, and it required individuals to create a total of five future non-smoking situations at different time points (one situation in a week, two situations in 2 weeks, one in a month, and one in 3 months) throughout the 8 weeks of treatment. The participants were encouraged to practice EFT situations a total of 98 times. The procedure followed for its implementation was as follows: (1) in the therapy session, the therapist asked the participants to identify a positive non-smoking event (e.g., a walk in the fresh air) that they eagerly hoped would happen within the proposed time frame. (2) The therapist asked the individuals to write the situation on a sheet of paper (including the place they had chosen, the company, activities, feelings, etc.) and to practice visualizing it for 2–3 min. (3) Subsequently, the participants were asked to rate the vividness of the situation on a 10-point scale. If they rated it below 6, the therapists helped the patients to improve the description of the situation to facilitate the visualization practice. As homework, patients had to practice visualization twice a day, as well as record the vividness of each practice. Participants did not receive any reminder (i.e., via phone or message) to perform the practice, but in the midweek sessions (or the B sessions) they were asked about the visualization practice, as well as whether they had doubts or questions related to the component. The total number of practices was the result of the number of times the person rated the vividness on the record sheet. Therefore, if the patient did not bring the sheet to the session, or the sheet was blank, even though they reported having performed the visualizations, the number of practices was considered to be zero.

CM is aimed at increasing substance abstinence rates by providing contingent reinforcers to the target behavior, usually substance abstinence. The efficacy of this therapeutic component is based on the increase in alternative reinforcers to drug use (Higgins, 1997). It is important to note that the effectiveness of this component is dependent on the magnitude and immediacy of the reinforcers given (Lussier et al., 2006). In this study, the CM component specifically consisted of providing incentives from the sixth session onwards in exchange for attaining tobacco abstinence, biochemically verified through CO ≤ 4 ppm and urine cotinine ≤ 80 ng/ml. In this sense, abstinent patients earned 20 points (equivalent to €20) in session 6, €25 in session 6B, €30 in session 7, €35 in 7B, and €40 in session 8 (i.e., post-treatment). In addition, for every two consecutive negative analyses, they would obtain a bonus of €10 extra in points. Therefore, a patient who had achieved tobacco abstinence could earn a maximum of €170. The article by Aonso-Diego et al. (2021) can be consulted for more information.

### Measures

During the intake session, sociodemographic information (e.g., sex), substance use variables (e.g., primary substance use), and smoking variables (e.g., cigarettes per day) were collected through an ad hoc questionnaire. Additionally, nicotine dependence was assessed using the Fagerström test for nicotine dependence (FTND) (Heatherton et al. 1991).

All participants provided a urine sample for cotinine and drug testing (cocaine, opioids, amphetamine, methamphetamine, and cannabis). They also provided a breath sample in order to evaluate CO and alcohol consumption.

The outcome variables were DD and cigarette demand. The DD task was evaluated using a computer program. Participants had to choose between receiving an immediate amount of money available now (between €5 and €995) and a fixed amount of money available later (€1,000). The program finds the indifference point for each of the five delays presented through the trials (i.e., 1 day, 1 week, 1 month, 6 months, and 1 year). The indifference point refers to the subjective value in which the delayed reward has an equivalent value to the immediate reward. In order to find the indifference point, an adjusting amount procedure was used in this task based on the one proposed by Holt et al. (2012). All participants were informed that the amounts of money presented were hypothetical, but that they should try to answer as realistically as possible. The total duration of the task does not usually exceed 10 min. The EFT cues were not presented while participants completed the DD task.

The CPT instructions are based on the original recommendations from MacKillop et al. (2008). Participants had to answer the following question: “How many cigarettes would you smoke if they were ____ each?” The following 19 prices were inserted: zero (free), € 0.01, € 0.02, € 0.05, € 0.10, € 0.25, € 0.50, € 1, € 2, € 3, € 4, € 5, € 10, € 20, € 50, € 100, € 250, € 500, and € 1,000. The prices were presented in ascending order. To complete the task, the participants were told to assume the following: (1) your income and savings are what you normally have, (2) the cigarettes are your favorite brand, (3) there is no other way to get cigarettes or nicotine, (4) if you buy none, you don’t smoke that day, (5) if you buy cigarettes, you must smoke them all on the same day, (6) cigarettes cannot be kept or given away, (7) your urge or desire to smoke is similar to how you feel today.

Both CPT and DD tasks as well as cotinine were evaluated eight times, once in the intake session and once a week in the midweek sessions. Furthermore, the total frequency of EFT practices during treatment was also recorded via self-report.

### Data analysis

Both the CPT and DD values were standardized and compared with a critical value of *Z* =  ± 4 in order to detect outliers (Tabachnick et al. 2001). If an outlier was detected, these values were recorded as the highest non-outlying value (plus 0.01 for AUC and plus 1 for the CPT indices). In addition, CPT and DD values were analyzed in order to detect nonsystematic data following the original recommendations of Stein et al. (2015) and Johnson and Bickel (2008). Two responses to the DD task were eliminated due to being considered nonsystematic. There were no nonsystematic responses in the CPT because the task was computerized, and the program alerted the user of inappropriate responses.

The area under the curve (AUC) was calculated according to Myerson et al. (2001), in order to analyze the indifference points of each participant. AUC values close to 0 indicate maximum DD rates, while values close to 1 indicate minimum DD rates.

Five demand indices were generated from the CPT, as follows: (1) intensity (cigarette smoking at zero cost); (2) O_max_ (maximum amount of money spent on cigarettes); (3) P_max_ (price associated with the maximum expense); (4) breakpoint (first price at which consumption was interrupted); (5) elasticity (proportional change in consumption based on the proportional change in price). Intensity, Omax, Pmax, and breakpoint were generated using an observed values approach. Elasticity was estimated using an exponentiated demand curve Eq. (1) (Koffarnus et al., 2015):

1 $$Q = {Q}_{0}0 \times {10}^{k (e-\alpha {Q}_{0}C-1)}$$

In Eq. (1), *Q* is consumption at commodity price C, *Q*_0_ is consumption at the minimum price, *k* is the range of consumption, and *α* is the elasticity of demand (i.e., the slope of the demand curve). Since 14% of the total CPT completed were classified as zero responders (60% in the last treatment session), it was decided to calculate the essential value (EV), which is inversely proportional to the *α* value, according to Eq. (2) proposed by Hursh and Roma (2016):

2 $$Essential Value = \frac{1}{100*\mathrm{\alpha }*{k}^{1.5}}$$

Since the value of *α* is impossible to estimate in zero responders, according to previous studies (Heckman et al., 2019; Stein et al., 2017; Yoon et al., 2021), the EV was defined as 0 (i.e., the lowest potential value).

The validity of the RP tasks was analyzed in two ways. On the one hand, the Pearson correlation was used to compare clinically relevant variables with the CPT and DD indices. On the other hand, a nonlinear regression was used to generate an *R*^2^ value to assess the goodness-of-fit of CPT.

Mixed-effects model repeated measures (MMRM) analysis with restricted maximum likelihood was used to explore whether the DD and CPT changes were due to treatment, number of EFT practices, or cotinine levels over time (in-treatment changes). An unstructured modeling of frequencies at each visit and within-subject error correlation structure was included in this analysis. Cotinine and the number of EFT practices were treated as time-varying covariates. The MMRM model allows us to analyze missing data from longitudinal studies (Vallejo et al. 2011). The statistical software used in this study was SPSS (v20, Chicago, IL).

## Results

### Correlations among the RP indices and smoking variables

As expected, cigarette demand and DD were related to some smoking variables, such as cigarettes per day. Furthermore, the CPT data of all participants presented a good fit in the exponentiated equation (median *R*^2^ = 0.97) (see Table 2).

**Table 2:** Correlations among smoking-related measures, delay discounting, and cigarette demand indices

	1	2	3	4	5	6	7	8	9	10
1—Cigarettes per day	-	-	-	-	-	-	-	-	-	-
2—Years of regular smoking	.15	-	-	-	-	-	-	-	-	-
3—Urine cotinine	.42**	.12	-	-	-	-	-	-	-	-
4—FTND	.72**	.30*	.32**	-	-	-	-	-	-	-
5—AUC	.37*	− .26	.10	.25	-	-	-	-	-	-
6—Intensity	.79**	.16	.43**	.59**	.25	-	-	-	-	-
7—Breakpoint	.16	− .09	.16	.11	.12	.11	.-	-	-	-
8—Omax	.28*	.08	.49**	.27*	.14	.27*	.74**	-	-	-
9—Pmax	.13	− .06	.19	.10	.08	.09	.97**	.75**	-	-
10—EV^1^	.32**	.14	.42**	.37**	.11	.30*	.34**	.68**	.32**	-

^1^Elasticity was estimated using essential value equation, so high values imply less elasticity. *EV*, essential value; *FTND*, Fagerström test for nicotine dependence; *AUC*, area under the curve of delay discounting task

^*^p ≤ 05; **p ≤ .01

### Effect of episodic future thinking on DD and cigarette demand

Overall, the participants performed the visualization tasks a mean of 33.227 (*SD* = 44.602) times with a mean vividness of 7.761 (*SD* = 1.420). Table 3 shows in detail the statistics of the EFT practice.

**Table 3:** EFT practice in intra-treatment sessions

Session	Frequency of EFT practice^a^	Vividness of EFT practice^a^
2	6.57 ± 5.69	7.77 ± 1.40
3	5.84 ± 6.55	7.62 ± 1.82
4	6.50 ± 9.29	7.79 ± 1.30
5	6.08 ± 10.02	7.99 ± 1.57
6	7.28 ± 12.45	8.23 ± 1.34
7	5.90 ± 12.45	8.09 ± 1.53

a mean ± *SD*, *EFT,* episodic future thinking

**Fig. 2:**
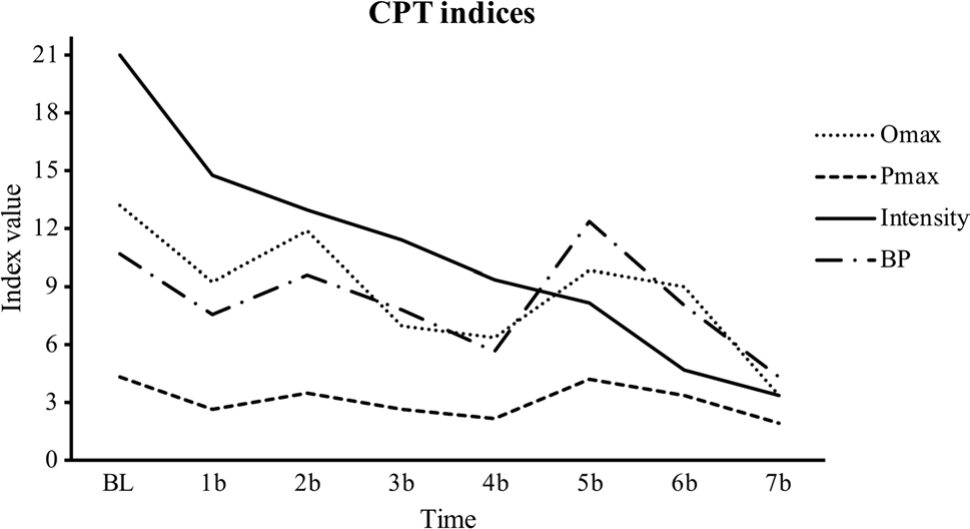
Evolution of observable CPT indices throughout treatment

**Fig. 3:**
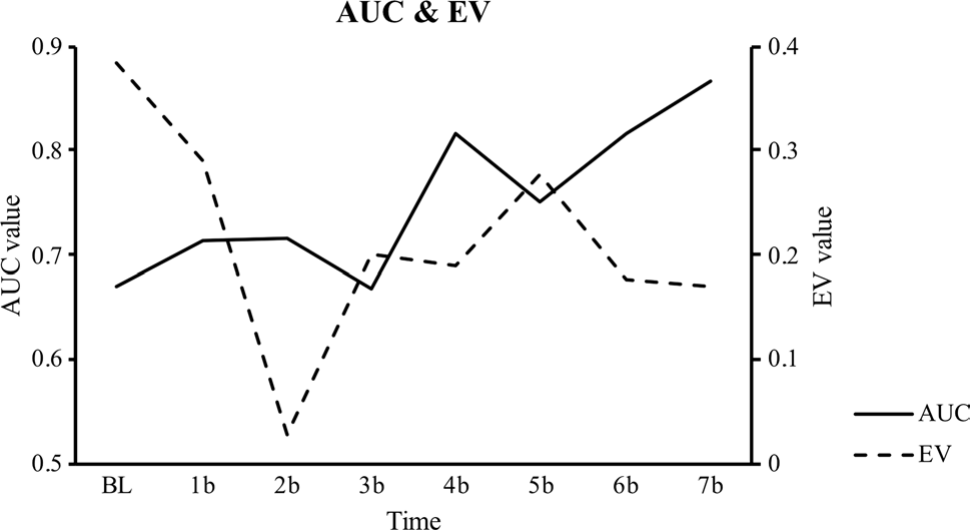
Evolution of AUC and EV throughout treatment

Tables 4, 5, 6, 7, 8, and 9 show the MMRM outcomes for each CPT index and AUC. The results show that the time effect was significant in all demand indices used (intensity, breakpoint, O_max_, P_max_, and EV), but not in the AUC. Figures 2 and 3 show the RP indices throughout the treatment sessions.

**Table 4:** Results of fitting taxonomy of MMRM models to the intensity

Fixed effect	Model A				Model B^1^				Model C			
	df_N_	df_D_	*F*	Pr > F	df_N_	df_D_	*F*	Pr > F	df_N_	df_D_	*F*	Pr > F
Time (β_1_)	7	35	33.486	.000	7	43	44.280	.000	7	8	7.679	.005
EFT (β_2_)	1	11	1.348	.271	1	1	.019	.907	1	12	.011	.919
EFT × time (β_3_)									7	6	.066	.504
EFT × COT (β_4_)					1	3	83.081	.003	1	5	4.318	.092
EFT × GRP (β_5_)					1	2	51.826	.024	1	2	2.440	.250
GRP (β_6_)	1	12	1.188	.297	1	20	9.920	.005	1	6	.489	.511
GRP × time (β_7_)					1	46	1.909	.090	7	9	1.937	.174
GRP × COT (β_8_)									1	7	.066	.805
COT (β_9_)	1	18	135.545	.000	1	3	310.716	.000	1	9	41.459	.000
COT × time (β_10_)									7	4	5.457	.060
Goodness-of-fit (AIC/BIC/parameters)												
	2258.1/2397.9/47				2237.0/2375.9/56				2335.4/2472.7/71			

^1^Information criteria allow us to conclude that model B provides a better fit than models A and C. *EFT*, number of episodic future thinking exercises practiced; *COT*, urine cotinine; *GRP*, treatment group; *df*_*N*_, numerator degrees of freedom; *df*_*D*_, denominator degrees of freedom; *AIC*, Akaike information criterion; *BIC*, Bayesian information criterion

**Table 5:** Results of fitting taxonomy of MMRM models to the breakpoint

Fixed effect	Model A				Model B^1^				Model C			
	df_N_	df_D_	*F*	Pr > F	df_N_	df_D_	*F*	Pr > F	df_N_	df_D_	*F*	Pr > F
Time (β_1_)	7	35	5.016	.001	7	25	4.272	.003	7	38	1.636	.155
EFT (β_2_)	1	24	12.246	.002	1	12	21.335	.001	1	58	.384	.538
EFT × time (β_3_)									7	36	1.176	.341
EFT × COT (β_4_)					1	29	3.249	.082	1	19	4.623	.045
EFT × GRP (β_5_)					1	18	1.294	.270	1	15	1.483	.243
GRP (β_6_)	1	24	3.650	.068	1	70	1.540	.270	1	75	.398	.530
GRP × time (β_7_)					7	24	.485	.836	7	40	.442	.870
GRP × COT (β_8_)									1	23	17.047	.000
COT (β_9_)	1	30	2.726	.109	1	33	.095	.760	1	38	1.515	.226
COT × time (β_10_)									7	19	6.005	.001
Goodness-of-fit (AIC/BIC/parameters)												
	2981.6/3121.4/47				2960.2/3099.1/56				3025.4/3162.7/71			

^1^Information criteria allow us to conclude that model B provides a better fit than models A and C. *EFT*, number of episodic future thinking exercises practiced; *COT*, urine cotinine; *GRP*, treatment group; *df*_*N*_, numerator degrees of freedom; *df*_*D*_, denominator degrees of freedom; *AIC*, Akaike information criterion; *BIC*, Bayesian information criterion

**Table 6:** Results of fitting taxonomy of MMRM models to the Omax

Fixed effect	Model A				Model B^1^				Model C			
	df_N_	df_D_	*F*	Pr > F	df_N_	df_D_	*F*	Pr > F	df_N_	df_D_	*F*	Pr > F
Time (β_1_)	7	29	14.336	.000	7	23	14.454	.000	7	18	3.403	.016
EFT (β_2_)	1	8	5.372	.049	1	7	4.010	.088	1	54	.072	.789
EFT × time (β_3_)									7	19	.972	.479
EFT × COT (β_4_)					1	19	7.529	.013	1	12	2.221	.161
EFT × GRP (β_5_)					1	13	2.890	.113	1	20	.185	.672
GRP (β_6_)	1	9	.014	.909	1	66	1.795	.185	1	79	.442	.508
GRP × time (β_7_)					7	23	1.601	.184	7	21	1.019	.447
GRP × COT (β_8_)									1	16	1.053	.320
COT (β_9_)	1	9	72.019	.000	1	14	27.525	.000	1	15	26.674	.000
COT × time (β_10_)									7	8	15.933	.000
Goodness-of-fit (AIC/BIC/parameters)												
	2772.5/2912.3/47				2747.4/2886.3/56				2806.2/2943.5/52			

^1^Information criteria allow us to conclude that model B provides a better fit than models A and C. *EFT*, number of episodic future thinking exercises practiced; *COT*, urine cotinine; *GRP*, treatment group; *df*_*N*_, numerator degrees of freedom; *df*_*D*_, denominator degrees of freedom; *AIC*, Akaike information criterion; *BIC*, Bayesian information criterion

**Table 7:** Results of fitting taxonomy of MMRM models to the Pmax

Fixed effect	Model A				Model B^1^				Model C			
	df_N_	df_D_	*F*	Pr > F	df_N_	df_D_	*F*	Pr > F	df_N_	df_D_	*F*	Pr > F
Time (β_1_)	7	35	3.647	.005	7	32	3.195	.011	7	35	1.819	.115
EFT (β_2_)	1	41	2.932	.094	1	35	6.099	.019	1	59	.627	.432
EFT × time (β_3_)									7	31	.812	.584
EFT × COT (β_4_)					1	86	.598	.441	1	74	1.119	.294
EFT × GRP (β_5_)					1	34	2.558	.119	1	39	3.242	.080
GRP (β_6_)	1	33	2.901	.098	1	78	1.751	.190	1	90	1.214	.274
GRP × time (β_7_)					7	34	.530	.805	7	32	.544	.794
GRP × COT (β_8_)									1	64	2.171	.146
COT (β_9_)	1	73	.558	.458	1	78	.094	.760	1	105	2.398	.124
COT × time (β_10_)									7	36	4.243	.002
Goodness-of-fit (AIC/BIC/parameters)												
	2357.2/2497.0/47				2350.6/2489.5/56				2442.4/2579.7/71			

^1^Information criteria allow us to conclude that model B provides a better fit than models A and C. *EFT*, number of episodic future thinking exercises practiced; *COT*, urine cotinine; *GRP*, treatment group; *df*_*N*_, numerator degrees of freedom; *df*_*D*_, denominator degrees of freedom; *AIC*, Akaike information criterion; *BIC*, Bayesian information criterion

**Table 8:** Results of fitting taxonomy of MMRM models to the EV (elasticity)

Fixed effect	Model A^1^				Model B				Model C^2^			
	df_N_	df_D_	*F*	Pr > F	df_N_	df_D_	*F*	Pr > F	df_N_	df_D_	*F*	Pr > F
Time (β_1_)	7	23	21.552	.000	7	21	22.323	.000	-	-	-	-
EFT (β_2_)	1	14	.000	.985	1	17	.023	.882	-	-	-	-
EFT × time (β_3_)									-	-	-	-
EFT × COT (β_4_)					1	23	.005	.943	-	-	-	-
EFT × GRP (β_5_)					1	9	.190	.673	-	-	-	-
GRP (β_6_)	1	15	2.635	.125	1	60	.715	.401	-	-	-	-
GRP × time (β_7_)					7	21	1.732	.156	-	-	-	-
GRP × COT (β_8_)									-	-	-	-
COT (β_9_)	1	17	.015	.904	1	27	.014	.908	-	-	-	-
COT × time (β_10_)									-	-	-	-
Goodness-of-fit (AIC/BIC/parameters)												
	− 3.9/135.1/47				43.1/181.2/56				2442.4/2579.7/71			

^1^Information criteria allow us to conclude that model A provides a better fit than models B and C. ^2^It was not possible to estimate model C due to a problem in the convergence of the model. *EFT*, number of episodic future thinking exercises practiced; *COT*, urine cotinine; *GRP*, treatment group; *df*_*N*_, numerator degrees of freedom; *df*_*D*_, denominator degrees of freedom; *AIC*, Akaike information criterion; *BIC*, Bayesian information criterion

**Table 9:** Results of fitting taxonomy of MMRM models to the AUC

Fixed effect	Model A				Model B^1^				Model C			
	df_N_	df_D_	*F*	Pr > F	df_N_	df_D_	*F*	Pr > F	df_N_	df_D_	*F*	Pr > F
Time (β_1_)	7	10	2.741	.074	7	14	2.393	.078	1	13	1.008	.468
EFT (β_2_)					1	8	17.551	.003				
COT (β_3_)									1	9	19.632	.002
COT × time (β_4_)									7	10	8.469	.002
Goodness-of-fit (AIC/BIC/parameters)												
	− 133.9/ − 23.0/44				− 134.0/ − 26.1/45				29.6/136.5/52			

^1^Information criteria allow us to conclude that model B provides a better fit than models A and C. It was not possible to create more complex models, as in CPT indices, due to a problem in the convergence of the models. *EFT*, number of episodic future thinking exercises practiced; *COT*, urine cotinine; *GRP*, treatment group; *df*_*N*_, numerator degrees of freedom; *df*_*D*_, denominator degrees of freedom; *AIC*, Akaike information criterion; *BIC*, Bayesian information criterion

Greater practice of the EFT component significantly reduced both the breakpoint (model B: β_2_ =  − 0.560837, *p* = 0.001) and the P_max_ (model B: β_2_ =  − 0.478805, *p* = 0.019) as well as the DD (model B: β_2_ = 0.019587, *p* = 0.003). Additionally, the EFT × COT interaction was significant both in intensity (model B: β_2_ = 0.0004879, *p* = 0.003) and in O_max_ (model B: β_2_ = 0.000323, *p* = 0.013), such that a greater reduction in cotinine levels coupled with greater EFT practice led to a greater decrease in both demand indices. On the other hand, EFT had no impact on EV (model A: *p* = 0.985).

### Effect of tobacco use reduction and treatment condition on DD and cigarette demand

The results showed that cotinine levels were significantly associated with some cigarette demand indices. Specifically, cotinine levels had a main effect on both intensity (model B: β_2_ = 0.004801, *p* < 0.001) and O_max_ (model B: β_2_ = 0.003244, *p* < 0.001). These results suggest that a greater reduction in cotinine, and therefore smoking consumption, was associated with a greater reduction in both demand indices.

On the other hand, the reduction of cotinine, especially in the first sessions of treatment (model C: β_4_ = 0.0001, *p* = 0.002), was related to a decrease in DD. Nevertheless, of note is that the model that best predicted changes in DD did not include cotinine as the main or interactive effect (see Table 6, model B).

Finally, participants of CBT + EFT reduced intensity less in comparison with CBT + EFT + CM, even when the participants practiced the EFT component more. (model B: β_2_ = 1.102479, *p* = 0.024).

## Discussion

The current study is the first to examine the effect of EFT on RP (DD and cigarette demand) during smoking cessation treatment in individuals with SUDs. Three main results have emerged: (1) greater self-reported practice of EFT reduced the two facets of RP; (2) tobacco use reductions decreased intensity of demand and O_max_; and (3) the addition of the CM component decreased the intensity of cigarette demand.

The number of EFT practices had an impact, either in isolation or interactively, on the two dimensions of RP (i.e., DD and cigarette demand). This outcome is explained by the effects of EFT which mainly consist of the expansion of the temporal window to subsequently produce an enhanced valuation of the future decisions (Snider et al., 2016). In this sense, EFT helps with the improvement of far-sighted decision-making, emotional regulation, prospective memory, and spatial navigation (Schacter et al., 2017). And ultimately, these functions of EFT can result in the initiation of healthy behaviors alternative to tobacco use, as well as in increasing the cost of smoking.

Regarding DD rates, these findings confirm and extend previous research in substance users (Bulley and Gullo 2017; Chiou and Wu 2017; Forster et al. 2021; Mellis et al. 2019; Patel and Amlung 2020; Snider et al. 2016; Sofis et al. 2020; Stein et al. 2016, 2018). Of note is that the short cues generated in EFT were not presented in the DD task. This is worth mentioning since a recent review has indicated that EFT decreases DD only when cues are present (Rung and Madden 2019). Furthermore, unlike several studies (see, e.g., Bulley and Gullo 2017; Chiou and Wu 2017; Mellis et al. 2019; Patel and Amlung 2020; Snider et al. 2016; Sofis et al. 2020; Stein et al. 2016), in the current one, the EFT delays (1 week, 2 weeks, 1 month, and 3 months), did not match the DD ones (1 day, 1 week, 1 month, 6 months, and 1 year). Despite previous research, this study indicates that these two conditions are not necessary to achieve a meaningful effect on DD.

An important and novel finding of the current study was that EFT also had a significant effect on the four observable indices of drug demand. Previous evidence has partially shown that EFT impacts on breakpoint (Patel and Amlung 2020), O_max_ (Voss et al. 2021), P_max_ (Patel and Amlung 2020), and especially on the intensity of demand (Athamneh et al. 2021; Bulley and Gullo 2017; Patel and Amlung 2020; Snider et al. 2016; Stein et al. 2018; Voss et al. 2021).

Several reasons may explain the effect of EFT on the two dimensions of RP. First, unlike other studies (see, e.g., Chiou and Wu 2017; Stein et al. 2016, 2018), this sample consists of treatment-seeking smokers, rather than current smokers without motivation to quit, a variable strongly related to RP (Sheffer et al. 2019; Veilleux and Skinner 2016). Second, the number of days of abstinence from substance use was higher (an average of 274.60), compared to previous EFT studies with SUD populations (see, e.g., Forster et al. 2021; Sofis et al. 2020; Voss et al. 2021). These differences in the participant profiles may have an impact on RP.

Taken together, these findings suggest that implementing an EFT component within a standard treatment for smoking cessation among smokers with SUDs could be useful for reducing RP, despite the difficulty of constructing specific future scenarios for the SUD population (see, e.g., D’Argembeau et al. 2006; El Haj et al. 2019; Mercuri et al. 2015, 2016, 2018; Moustafa et al. 2018). This may explain the number of EFT practices required (14 every week for 7 weeks) since this population needs intensive treatments in terms of the number of sessions and the time dedicated (Murphy and McKay 2004; Richter and Arnsten 2006; Schroeder and Morris 2010). Furthermore, several articles have recently pointed out the need to continue EFT practice to achieve a significant effect on RP (Mellis et al. 2019; Patel and Amlung 2020).

Consistent with the previous literature (see, e.g., Higgins et al. 2018; Nighbor et al. 2020; Smith et al. 2017; Streck et al. 2018; Weidberg et al. 2018), the reduction in tobacco use resulted in a decrease in cigarette demand, specifically in O_max_ and intensity of demand. Furthermore, this finding is congruent with others indicating that individuals with greater tobacco use show a higher cigarette demand (González-Roz et al. 2019). This result is aligned with the theory of relative reinforcing efficacy (Bickel et al. 2000), which highlights that an increase in cigarette cost, defined inclusively to encompass the monetary cost, effort, or time required to acquire cigarettes (Bickel et al. 2014), will produce a reduction in the reinforcing value of nicotine. Thus, nicotine fading along with other treatment components may reduce the motivation to smoke (Murphy et al. 2017) and, therefore, increase the cost of smoking behavior.

Finally, CM was associated with a greater reduction in the intensity of demand, as was found in Weidberg et al. (2018). It is likely that the increase in the availability of alternative reinforcers to tobacco use provided by CM (Higgins et al. 2008; Stonerock and Blumenthal 2017) yielded a decrease in the reinforcing value of nicotine, i.e., in cigarette demand. This result also points in the same direction as the meta-analysis by Acuff et al. (2020), where it is highlighted that introducing an opportunity cost could reduce the intensity of demand by altering the motivation to consume a substance.

At the clinical level, considering that changes in cigarette demand have been related to both short- and medium-term tobacco abstinence (Madden and Kalman 2010; Murphy et al. 2017), the current findings add support to the use of behavioral strategies based on nicotine fading and the implementation of incentives that reinforce abstinence, as effective interventions for smoking cessation.

This study is not exempt from limitations, which are detailed below. First, the sample used was relatively small and this limited the statistical analyses performed and the complexity of interpreting the models tested. Despite this, the present work found several significant effects of relevance, and with regard to the target population (i.e., smokers with SUD), other published articles present a similar number of participants or fewer (Alessi et al. 2008; Alessi and Petry 2014; Cooney et al. 2017). Second, this work did not include a control group (i.e., CBT or EFT only), which would increase the strength of the results regarding the impact of EFT on RP. Furthermore, of note is that the number of EFT practices was self-reported, so it is not exempt from all the related biases.

In summary, the current study enhances the available knowledge about the effectiveness of EFT in reducing RP, both in terms of cigarette demand and DD. In addition, the results add support for the use of behavioral interventions (e.g., nicotine fading) in the treatment for smoking cessation among individuals with SUDs. Future studies should further explore the usefulness of EFT in the treatment of SUDs in clinical settings.
